# The Perfect Man

**Published:** 1875-03

**Authors:** 


### THE PERFECT MAN.

In forming a conception of the man who shall represent the standard of physical perfection, we may be aided by consulting the regulations of our own and other governments as to the enlistment of men for military service. Stature, weight, and circumference of the chest, are the leading considerations. The relative proportions of these are approximately established. There is no fixed rule as to the maximum height of the recruit. Very tall men are most effectual in military display, and are always the pride of their country when on dress-parade. But tested by their ability to endure the fatigue and hardship of active service, they are inferior to men of medium height. The medical examiner will make a careful inspection before he "passes" the candidate who stands "6 feet 2." Very likely this noble stature is attended with serious defect in the development of some part of the body. Perhaps his chest is contracted, and his muscles soft and flaccid. He excels in length of legs, but in other respects is deficient.

The minimum height of the recruit is fixed by our army regulations at 5 feet 3 inches. In the English army it is 5 feet 5 inches; and in the French, 5 feet 1 inch. If we take the minimum of the United States Army regulations, 5 feet 3, and the unofficial maximum of 6 feet 2, which the experience of medical men suggests, we have a medium of 5 feet 8½ inches. In a list of more than 1400 English soldiers, 78 per cent. were below this medium. Of 1000 French soldiers, 91 per cent. were below; while of 600 men, representing regiments from different parts of the Union, and the various nationalities to be found in our army, but 75 per cent. were less than 5 feet 8½. The records of one of our recruiting stations for three years, in a time of peace, show that the medium height of the men of American birth was a fraction over 5 feet 8 inches.

There are no rules limiting in either direction the weight of enlisted men. Dr. Bartholow, of the U. S. Army, whose "Manual of Instructions," adopted by the Surgeon-General, affords much of the material for this article, declares that recruits of the minimum height, 5 feet 3 inches, should weigh not less than 120 pounds. Surgeon-General Hammond (now Professor Hammond, of Bellevue Medical College), allows as a just proportion, five pounds to every additional inch above 5 feet 5 inches. If we extend this rule to 5 feet 3, the regulation minimum, and apply it to Bartholow's proposed minimum weight of 120 pounds, we have for our soldiers of 5 feet 8, a weight of 145 pounds. With reference to maximum weight, Bartholow says, "No man weighing more than 220 pounds is fit for active military service." We would suggest that no man of that weight is fit for *any* "active service;" and, as a military standard, our maximum would be much below those figures.

The capacity of the chest affords a valuable indication of the power and vitality of the system. It is found that a healthy man of 5 feet and 8 inches in height, expires after the deepest inspiration 233 cubic inches of air. To every inch of increased stature there should be added a lung-capacity of eight cubic inches. But, in general observation, it is not practicable to measure the capacity of the lungs in cubic inches. Therefore, we will consider the circumference and mobility of the chest as ascertained by the tape-measure. In 150 soldiers, the height ranged from 5 feet 4½ inches to 5 feet 11½. The chest measured from 29½ to 38 inches, the mean circumference being nearly 34 inches. In this list the tallest man had the least girth of chest, while a man 5 feet 8½ inches, possessed the greatest. For every inch in height there should be an increase of half an inch in the chest. No man should be accepted as a recruit with a chest-circumference of less than 30 inches. Hammond's rule is that the chest should measure half the height. This gives to the man of 5 feet 8 inches a chest of 34 inches girth. The difference in the chest when the lungs are filled with air after a deep inspiration and when they are exhausted by a forced expiration, is called the mobility. This differs from 2 to 5 inches. Less than two inches indicates disease. The medium mobility is 3 inches. In measuring the chest the tape should be applied immediately over the nipples. This is Hammond's rule; others say just *above* the nipples.

Now, if from the foregoing data we construct our model man, he will stand 5 feet and 8 inches in height, will weigh 145 pounds, and will show a chest of 34 inches circumference, with at least 3 inches mobility, and a capacity of 233 cubic inches. He will breathe 18 or 20 times in a minute, and his pulse will be about 75 and regular in rythm and force. He will stand upright, and walk with his shoulders well thrown back. Such a man must blame himself if he does not live to a good old age.

